# Calves disbudded with local nerve block and analgesic show conditioned place aversion two days later but not in the hours post-disbudding

**DOI:** 10.1017/awf.2026.10082

**Published:** 2026-04-20

**Authors:** Elizabeth Miriam Ledger, Thomas Ede, Michael Mendl, Benjamin Lecorps

**Affiliations:** 1 Bristol Veterinary School, United Kingdom; 2 University of Pennsylvania, United States

**Keywords:** Affective state, animal welfare, conditioned place preference, dairy cattle, dehorning, emotions

## Abstract

Hot-iron disbudding is a very common, painful procedure performed in dairy farms. One of the gold standard practices recommends combining the use of a local anaesthetic (e.g. procaine) and analgesic (e.g. meloxicam) to control pain. However, it is unknown if calves still experience pain during and after the procedure when using multi-modal pain relief. Here, we explored the affective consequence of disbudding using a conditioned place aversion paradigm where inferences are based on learnt aversion to places associated with negative experiences. We conducted two experiments: (1) calves were disbudded in their home-pen and then conditioned immediately afterwards for 6 h so that conditioning involved post-operative pain only; and (2) calves were disbudded in the conditioning compartment and remained there for the following 6 h so that conditioning included the potential pain and fear from the procedure and any post-operative pain. All calves were conditioned in the other (control) conditioning compartment either 2 days before or after disbudding. In both experiments, calves who were disbudded on the second conditioning (control conditioning happening 2 days before the procedure) showed no aversion to the compartment associated with disbudding, suggesting that pain was minimal in the 6 h post-disbudding. However, in Experiment 2, calves displayed a preference for the disbudding compartment when disbudding occurred first (control conditioning happened 2 days later) suggesting they were in more pain on day 2 than in the hours following the procedure. These results show that calves may experience pain for days after hot-iron disbudding, calling for more work on long-lasting pain following disbudding.

## Introduction

Hot-iron disbudding is a routine farm procedure that aims to remove horn buds and prevent future horn growth by destroying horn bud tissues (Stafford & Mellor 2005). Despite the perceived safety benefits of disbudding for other livestock and people (Knierim *et al.* 2015), the procedure leads to a range of negative welfare outcomes, the main one being the acute and potentially persistent experience of pain. Currently, one of the gold standards for disbudding calves involves the combined action of a local block (e.g. procaine), which prevents calves from feeling the pain during disbudding (if fully efficient), and an analgesic (e.g. meloxicam), which aims to control the post-operative pain in the hours after the procedure (Winder *et al.* 2018).

Although assessing animal pain is challenging, various approaches have been used to assess disbudding pain, including measures of wound sensitivity (Reedman *et al.* 2022), physiological markers of stress, such as cortisol and heart rate (Heinrich *et al.* 2009), and spontaneous behavioural changes, such as ear flicks (Faulkner & Weary 2000). However, all of these measures allow only limited inferences to be made regarding the affective component of pain (Weary *et al*. 2017; Ede *et al.* 2019b).

The affective component of pain is both affected by and contributes to learning and decision-making (Bateson 1991). This has led to the development of alternative measures that provide better assessment of felt emotions and subjective experiences, such as judgment bias tests (Harding *et al.* 2004; Neave *et al*. 2013; Daros *et al*. 2014; Lecorps *et al*. 2019), anhedonia tests (Lecorps *et al*. 2020), and learnt aversion paradigms, such as conditioned place aversion (CPA) (Ede *et al*. 2019a). The latter are based on associations that animals make between an affective experience (e.g. pain) and the environment in which it was experienced. For instance, a previous study has shown calves develop an aversion for a place where they were disbudded, compared to where they were just sedated (Ede *et al*. 2019a). Although previous studies showed that calves prefer a place where they received additional pain control (especially meloxicam) after disbudding (Ede *et al*. 2019c), indicating the drug provides some pain relief, it is not known whether calves still experience pain in the hours after disbudding when given analgesics. Here, we aimed to explore whether calves still found disbudding aversive despite the use of multi-modal pain relief using CPA. To assess whether calves experience residual pain in the hours after disbudding, we first (Experiment 1) explored whether they would show an aversion to a compartment associated with the 6 h after disbudding (using a local anaesthetic and analgesic). In this experiment, calves were disbudded in their home-pen directly before being moved to the conditioning compartment for 6 h. We expected calves to display an aversion to the compartment associated with this experience if the combined action of the local nerve block and the analgesic did not significantly mitigate the post-operative pain.

Furthermore, when effective, local anaesthetics and analgesics only control for pain and do not prevent animals from experiencing other negative emotions such as fear, which may result from human handling and injections (Ede *et al.* 2018; Arkangel 2023). Hence, a second study (Experiment 2) explored whether calves would display an aversion to the full procedure (accounting for both pain and fear), plus the following 6 h. In this experiment, conditioning included both the procedure (disbudding with local anaesthetic and analgesic) and the 6 h recovery period, similar to previous studies (Ede *et al.* 2019a,c, 2020). We expected calves in Experiment 2 to display an aversion to the disbudding compartment because of the combined action of fear and remaining pain.

There is limited understanding of the longer-term pain caused by disbudding. While some evidence suggests that pain may not extend beyond 24 h (Faulkner & Weary 2000; Weary *et al*. 2017; Lecorps *et al*. 2019) others suggest pain may last for days (Mirra *et al*. 2018; Casoni *et al*. 2019; Lecorps *et al*. 2020) or weeks (Adcock *et al*. 2020). Given that meloxicam’s analgesic properties likely wane after 22 h (Stock & Coetzee 2015), it is possible that calves may be more in pain on the second day after disbudding. As balancing treatment order is necessary to control for order effects in CPA (Ede *et al*. 2019a,c, 2020), we also expected that calves’ aversion may be influenced by the order in which they experienced the post-disbudding conditioning session and the control conditioning session. Calves who were disbudded first (and consequently experienced the control conditioning 2 days after disbudding, when residual pain may still exist) were expected to show a lower aversion for the disbudding compartment compared to those who were disbudded second (and consequently experienced the control treatment 2 days before disbudding, when they were not in pain).

## Materials and methods

### Ethical approval

This study was approved by The University of Bristol AWERB (Animal Welfare Ethical Review Board) Committee (# UIN/23/072). No calves were disbudded for the purpose of this study alone to avoid imposing unnecessary harms consistent with the 3Rs principles. This set of experiments was designed using the PREPARE and ARRIVE guidelines consistent with best practices.

### Study animals and housing

Calves were housed at Bristol University’s dairy farm in accordance with its standard husbandry practices. They were fed 4 L of colostrum (Brix value > 22%) within 6 h of birth, and then another 4 L 12 h later. Separation from the dam occurred as early as possible after birth. Calves were then housed in pairs in hutches (2.5 × 1.5 m; length × width) bedded with straw with free access to a fenced outdoor space measuring 1.6 × 1.5 m (Calf Igloo, Maghera, Northern Ireland, UK). Calves were fed 2.8 L of milk replacer (Sprint Plus 50, Bridgmans Farm Direct, Shepton Mallet, UK; 190 g L^–1^) from buckets with attached teats (Calf Feeding Teat Bucket 8L, Kerbl UK Limited, Rutland, UK) twice per day (approximately 0700 and 1630h) for the first 10 days, followed by 3 L per meal for 4 weeks. Then, calves were moved to groups of 4 (in 2.5 × 2.3 m hutches with 3.1 × 2.9 m of fenced outdoor space; Calf Igloo, Maghera, Northern Ireland, UK) where they were fed 3.5 L of milk replacer per meal from row feeders (5 Teat Calf Feeder, Wydale Plastics Limited, Crewkerne, UK) twice per day until weaning. Calves were given *ad libitum* access to water, straw (a mixture of wheat and barley), and grain (18% Premium Calf, Tamar Milling, Whitstone, UK). The outdoor fenced area was cleaned, and fresh straw was added to hutches daily. After the period of enrolment in our experiment (5 days), calves either stayed to become dairy cows or were sold to beef farms.

This study used 42 calves (20 in Experiment 1, 22 in Experiment 2, see *Statistical analysis* for details on power analysis), including a mix of different breeds (Experiment 1: 2 British Blue × Holstein heifers, 6 British Blue × Holstein bulls, 3 Holstein heifers, 7 Longhorn × Holstein bulls, and 2 Longhorn × Holstein heifers; Experiment 2: 3 British Blue × Holstein heifer, 4 British Blue × Holstein bulls, 2 Holstein heifers, 8 Longhorn × Holstein bulls, and 5 Longhorn × Holstein heifers). This sample is representative of calves raised on dairy farms that now often include beef-crossed breeds alongside dairy breeds. We were unable to balance breed and sex across both experiments due to the practical constraints of conducting research on a commercial dairy farm. Data were collected between May and December 2024. The mean (± SD) age of calves at enrolment was 23 (± 4.92) days of age. Five calves were not included in the analysis (Experiment 1: 1 calf, Experiment 2: 4 calves) due to technical issues and sickness, leaving a total of n = 19 for Experiment 1 and n = 18 for Experiment 2.

### Experimental procedure

The two experiments differed only in terms of whether calves experienced disbudding in their home pen (Experiment 1) or in the conditioning compartment (Experiment 2).

### Study apparatus

**Figure 1.**
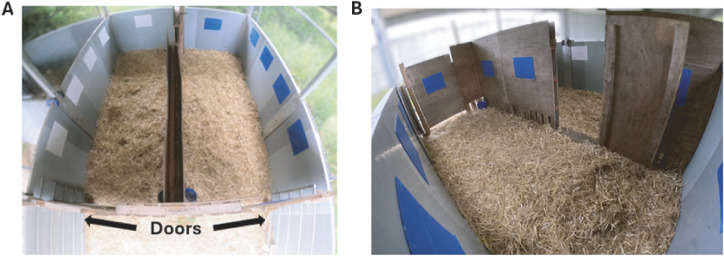
Conditioned Place Aversion apparatus composed of two identical compartments (3.7 × 1.5 m; length × width) with either blue or white squares (A) connected by a door in the middle partition (B). Calves entered the apparatus from the starting box (2.75 × 2.55 m) in front of the two compartments giving them access to both compartments via two separate doors.

The apparatus was adapted from previous studies (e.g. Ede *et al.* 2019a) and was made of two identical compartments (3.7 × 1.5 m; length × width) connected by a door. Calves entered the starting box (2.75 × 2.55 m) in front of the two compartments giving them access to both compartments via two separate doors, allowing them a choice as to which compartment to enter first (Figure 1). Visual cues made of white (left) and blue (right) rectangles were placed to enhance the association between the compartment and the different treatments. Although previous studies used blue and red panels (Ede *et al.* 2019a), a persistent colour bias was shown in recent work (Lafon *et al.* 2024) which was addressed when using blue and white panels (Hendricks *et al.* 2026).

Treatment order was pseudo-randomly allocated within experiments. Both experiments involved three phases: habituation; conditioning (two treatments: control and disbudding, which varied between the two groups as described below); and testing.

### Habituation

**Figure 2.**
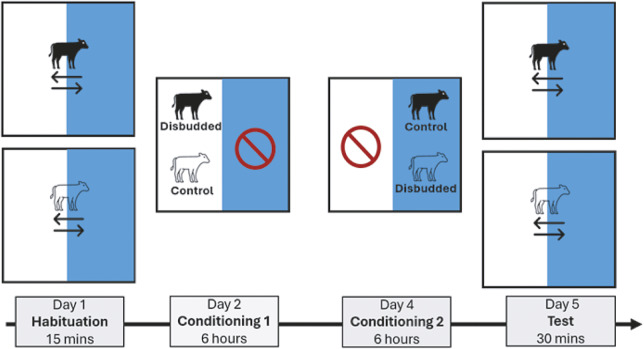
Experimental timeline. All calves were habituated individually with both compartments accessible on day 1. On day 2, calves were conditioned to either disbudding or nothing (control) in pairs in either compartment. On day 4, calves were conditioned in the opposite compartment (opposite side and colour), with the opposite treatment – pairs remained constant. On day 5, all calves were tested alone to assess conditioned place aversion by letting calves roam freely between the two compartments. The figure represents 2 calves enrolled in either Experiment 1 or 2. For each experiment, a second pair was run simultaneously but in the opposite compartments. The calf icon was reproduced from The Noun Project (2026).

On the day of enrolment, all calves were habituated to the arena individually by allowing them to explore the 2 compartments freely for 15 min (Day 1, see Figure 2). All three doors of the arena were opened so that calves placed in the starting box could enter either the blue or white compartments and then navigate between compartments. The colour panels of the compartment, as well as the inside of the pens, were visible to the calves when they made their choice. The 15-min time to explore the pens began once the calf had put both front feet into one of the pens, and the doors connected to the start box were then closed. Fifteen minutes was chosen to minimise social stress while still allowing all calves to explore both compartments.

The first compartment entered, and the time spent in each compartment was recorded using a mounted GoPro 10 (GoPro, USA), placed above the apparatus to give a full view of both compartments (see Figure 1). All calves explored both compartments during habituation.

### Conditioning

The compartment associated with the 6 h following disbudding only (Experiment 1) or disbudding and the 6 h following the procedure (Experiment 2) was pseudo-randomly assigned and counterbalanced between calves. Treatment compartment assignment was counterbalanced with the pre-treatment preference displayed during habituation (based on time spent in each compartment) so that approximately half of the calves had the disbudding treatment in their preferred compartment, and the other half received the control treatment in this compartment similar to previous studies (Ede *et al.* 2019a,c, 2020). All efforts were made to counterbalance initial preferences while keeping familiar calves together during conditioning. All conditioning was performed in pairs such that for each pair, one calf experienced the disbudding treatment, while the other experienced the control treatment. This was reversed for the second conditioning session so that calves were in the opposite side and colour compartment when receiving the second treatment.

During conditioning, calves were provided with *ad libitum* straw and water access. The calves were conditioned in familiar pairs (housed in the same pen) and disruption to housing and other pen mates was minimised before and during the experiments. We used 6-h conditioning to be consistent with previous studies (Ede *et al.* 2019a,c, 2020). During this time the local nerve block typically wanes while pain relief is only provided by the analgesic. In both experiments, 4 calves were run simultaneously, 1 of each treatment in each compartment. While calves could not see into the opposite compartments, as per other studies using similar experimental designs (e.g. Ede *et al*. 2019a), it is possible that they could hear other calves in the opposite compartment.

### Experiment 1

Nineteen calves were conditioned to associate only the post-operative pain with one of the CPA compartments. For this, calves were disbudded in their home pen and then immediately moved to one of the CPA compartments in pairs for the following 6 h. They were then returned to their home pen. This was done to prevent any associations between the conditioning compartments and the disbudding procedure. All calves were also conditioned either in absence of pain (two days before disbudding) or in expected lower pain (two days after disbudding). We elected to avoid any additional restraint handling and sham injections in the home pen during this control conditioning because these may have led to negative affect, especially in recently disbudded calves. Control and disbudding conditioning sessions took place on Days 2 and 4, counterbalanced for order across calves (Figure 2).

### Experiment 2

Eighteen calves were conditioned to associate both the procedure (pain and fear of procedure) and any post-operative pain experienced in the 6 h after the procedure with one of the CPA compartments. This was achieved by moving calves to one of the CPA compartments immediately before disbudding occurred (see below for disbudding procedure). The calves then remained in one of the conditioning compartments for the 6 h following the procedure before returning to their home pen. All calves were also conditioned either in absence of pain (two days before disbudding) or in expected lower pain (two days after disbudding). During control conditioning, calves were gently moved to one of the conditioning compartments and were neither handled nor given sham injections to prevent any unwanted negative associations. However, because calves were in pairs so that one calf experienced the control conditioning while the other experienced the conditioning associated with disbudding, control calves witnessed another calf being disbudded before the pair was left in the compartment for the remaining 6 h. Control and disbudding conditioning sessions took place on Days 2 and 4, counterbalanced for order across calves (Figure 2).

### Testing

On the morning of Day 5 (Figure 2), approximately 24 h after the beginning of the last conditioning, calves’ aversion for one of the two compartments was assessed by letting each calf individually roam freely between the 2 conditioning compartments (in the same manner as habituation) for a duration of 30 min, in line with a previous study (Lafon *et al.* 2024). The calf was placed into the start box so that they were able to choose a compartment. The 30 min began once the calf had placed both front feet into one compartment and the doors leading to the compartments were then closed. Aversion to either compartment was assessed by recording the first compartment entered (disbudding compartment or control compartment), the time spent in each compartment and where calves chose to lie down (if they did). Blinding was applied to treatment so that the observer did not know in which compartment calves were conditioned when watching videos. The order in which the calves were tested was randomised.

### Disbudding procedure

Disbudding was performed using a hot iron, (No 135 Alios gas disbudder, maximum temperature of 650°C as stated by manufacturer (Guilbert Express, France), pre-heated for at least 5 min). Calves were administered local anaesthesia in the form of a cornual nerve block (procaine hydrochloride 5%, 5ml per horn bud) and analgesia using a subcutaneous injection of meloxicam (0.5 mg kg^–1^), approximately 5 min prior to disbudding. We waited at least 10 min between doses to ensure the anaesthetic had time to act before administering more procaine (2.5 ml) if a calf still reacted to a needle prick test (7 calves in experiment 1 and 10 calves in experiment 2). Two calves were disbudded at a time. All calves who were being disbudded were restrained with a halter during injections and disbudding.

### Statistical analysis

All statistical analyses were performed in RStudio (RStudio Team 2024). Sample size calculation was done with the power.t.test function from R, for an alpha level of 0.05 and 0.8 power. Based on results from Ede *et al*. (2019a), we used a delta of 282.1 and standard deviation of 167.9 (first aversion test, comparison of time spent in disbudding and sham pens). The calculated minimum sample size was 7 calves per treatment group, but we chose to increase the sample size to at least 9 as we expected smaller effect sizes. The R script and data are shared in the Supplementary material.

Given the nature of our apparatus, the time calves spent in one compartment was directly related to the time they spent in the other one, so we only focused on time spent in the compartment associated with recovery from disbudding (Experiment 1) and disbudding plus recovery (Experiment 2). For analysis, time spent in this compartment was compared to what would be expected by chance (i.e. a 50/50 distribution between the two treatment compartments) by subtracting half the test time (i.e. 15 min) and testing its significance from zero.

Differences from chance in time spent in the disbudding pen were analysed using linear models (Bates *et al.* 2015). First, full models were conducted testing all fixed effects together: colour (blue or white); disbudding treatment order (first or second); time spent in the disbudding compartment during habituation; breed (Holstein, Longhorn or British Blue); and sex (male or female). Normality and homoscedasticity of residuals were confirmed graphically. Simplified models including only tendency/significant factors (*P* < 0.1) from the full model were then conducted. If no fixed effect was significant, the simplified model was a null model. Fit of the full and simplified models was compared by ANOVA, and results from both models are reported. Treatment effects (disbudded or not) were interpreted by comparing model intercepts (i.e. estimated mean of the difference between time in the disbudding compartment and what would be expected by chance) to the null expectation (0). In the case of significant factors, intercepts were compared to 0 for each level of a significant factor. In addition, because we expected order to affect CPA, we ran *t*-tests at each level (disbudded first or second) for each experiment, regardless of whether the order effect was significant in the full model. We used Bayesian statistics (BayesFactor package; Morey & Rouder 2023) to interpret non-significant effects.

Calves’ first compartment entered (disbudding or control), and where they first lay down (control or disbudding compartment) were analysed with a Chi-squared test (R’s basic Chi-squared test function). Difference from chance in the time spent in the disbudding compartment was directly compared between experiments with a Welch two sample test using R’s *t*-test function.

## Results

### Experiment 1

**Figure 3.**
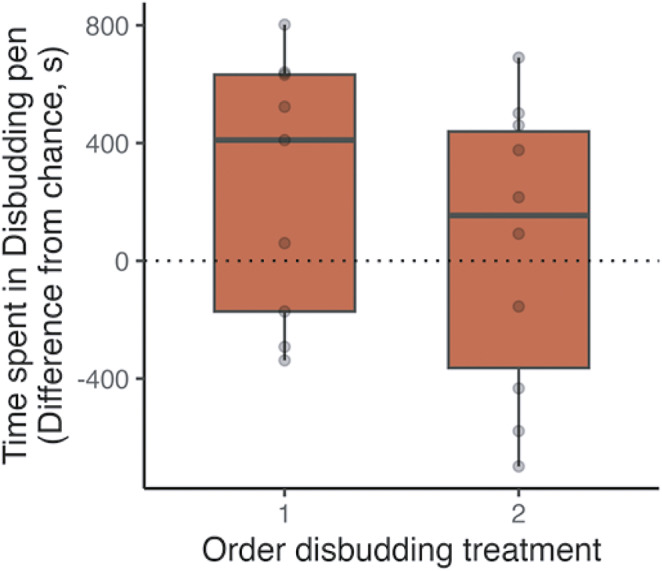
Conditioned Place Aversion results for Experiment 1. Time (s) different from chance spent by calves (n = 19) in the disbudding compartment during testing according to their treatment order: (1) disbudding first, control second; (2) control first, disbudding second. Time 0 corresponds to 50% (900 s) of the test duration. Grey circles represent individual points.

Colour, order, breed, sex and time spent during habituation did not have effects on the time spent in the disbudding compartment (all *P*s > 0.1). The full model did not improve fit compared to the simplified model (F_6,12_ = 0.75; *P* = 0.62). In the simplified model, time spent in the disbudding compartment did not differ from what would be expected by chance (mean [± SD] 144.0 [± 106.8] s, t_18_ = 1.3; *P* = 0.19; Order 1: 251.7 [± 441.1] s, Order 2: 47.1 [± 488.3] s; Figure 3). However, the Bayesian factor was low (BF01 = 1.93) indicating that the data are inconclusive and care should be taken in rejecting the alternative hypothesis. Furthermore, calves disbudded first (t_8_ = 1.71; *P* = 0.13) and second (t_9_ = 0.31; *P* = 0.77) did not avoid the compartment associated with the post-operative pain. For calves disbudded first, the Bayesian analysis (BF_01_ = 1.07) indicated that the data are inconclusive so care should be taken in rejecting the alternative hypothesis. For calves disbudded second, the Bayesian analysis (BF_01_ = 3.11) supports the absence of difference.

There was no significant difference in the compartment that was first entered (X^2^ [1] = 0.47; *P* = 0.49), or in which compartment the calves chose to lay down (X^2^ [1] = 0; *P* = 1) (Figure 1, supplementary material).

### Experiment 2

**Figure 4.**
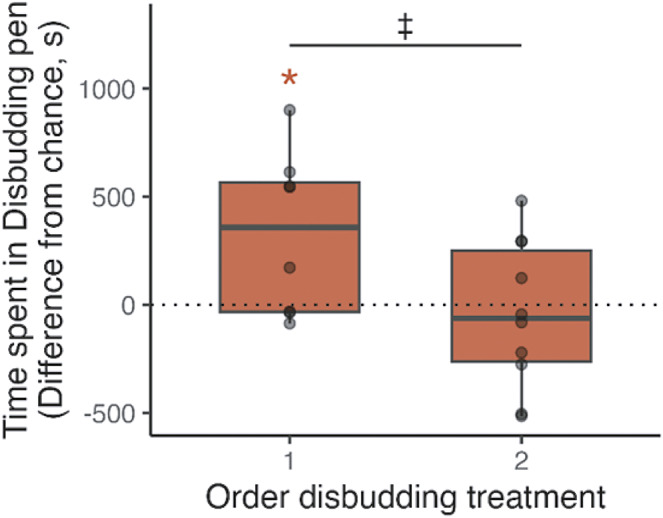
Conditioned Place Aversion results for Experiment 2. Time (s) different from chance spent by calves (n = 18) in the disbudding compartment during testing according to their treatment order: 1 disbudding first, control second, 2 control first, disbudding second. Time 0 corresponds to 50% (900 s) of the test duration. Grey circles represent individual points.

Colour, breed, sex and time spent during habituation did not have an effect on the time spent in the disbudding compartment (all *P*s > 0.1). However, order of disbudding tended to affect calves’ preferences, with calves disbudded first spending more time in the disbudding compartment than when disbudded second (397.6 [± 196.7] s, t_6,11_ = –2.0; *P* = 0.068). The full model did not improve fit compared to the simplified model (F_6,11_ = 0.34; *P* = 0.88). In the simplified model, disbudding order was significant (t_1,16_ = –2.2; *P* = 0.04), where calves disbudded first spent more time than would be expected by chance in the disbudding compartment during testing (328.3 [± 125.4] s, t_7_ = 2.6; *P* = 0.04). Calves disbudded second did not display an aversion for either compartment (–44.6 [± 112.2] s, t_9_ = –0.4; *P =* 0.70*)* (Figure 4), with good evidence of no difference (BF_01_ = 3.01).

There was no difference in the compartment that was first entered (X^2^ [1] = 0.22; *P* = 0.64), or in which compartment the calves chose to lay down (X^2^ [1] = 0.29; *P* = 0.59). (Figure 2, supplementary material).

## Discussion

This study explored whether a local nerve block and analgesic significantly reduce the pain experienced by calves during disbudding and in the 6 h following the procedure, to a level that is not detectable using CPA. In both experiments, calves who were disbudded second showed no aversion to the disbudding compartment, which is consistent with the idea that they did not experience detectable pain during or in the hours following disbudding. However, in Experiment 2, calves who were disbudded first showed a conditioned preference for the compartment in which they were disbudded.

Calves disbudded second had a true ‘no pain’ experience during the first control treatment (2 days before disbudding). These calves did not display an aversion for where they experienced the post-operative pain alone (Experiment 1) or were disbudded (Experiment 2) suggesting that a local nerve block and analgesic is effective in controlling the pain during and in the 6 h post-disbudding. Bayesian analyses further support the absence of difference in these calves. It has previously been difficult to determine whether the combination of local anaesthesia and analgesia fully eliminates or just reduces pain post-disbudding, with many measures used, and no clear consensus on duration of pain mitigation (Herskin & Nielsen 2018; Winder *et al.* 2018). Using CPA Ede *et al.* (2019a) showed that calves disbudded under sedation and local anaesthesia without post-operative analgesia displayed an aversion to the disbudding compartment, and further work then showed calves preferred the compartment associated with meloxicam compared to only a local block (Ede *et al.* 2019c), demonstrating that meloxicam reduces pain in the hours following disbudding. However, when comparing hot iron and caustic paste disbudding (using a sedative, a local nerve block, and meloxicam), Ede *et al.* (2020) showed a higher aversion for the compartment associated with caustic paste disbudding suggesting calves were in pain despite the use of meloxicam when using caustic paste. Our results build on these findings, suggesting meloxicam may suppress the pain associated with hot-iron disbudding to a level below that which is detectable using CPA.

While calves disbudded second showed no aversion to the disbudding compartment, calves disbudded first displayed a significant preference for the disbudding compartment in Experiment 2 (as measured via time spent in the compartment but no preference was detected for first entry nor where calves chose to lie down). Although unexpected, this order effect supports our prediction that calves may have been in pain on the second day after disbudding, consistent with the literature suggesting that meloxicam may not be effective beyond the first day post-disbudding. The elimination half-life of meloxicam injected subcutaneously (0.5 mg kg^–1^) is debated with estimates ranging from 22 (± 3) h in cattle (Stock & Coetzee 2015), 16.2 h in 7–8 month old calves (Meléndez *et al.* 2019), and longer estimates in pre-ruminant calves: 36.3 (± 21.7) h (calves ill with diarrhoea; Shock *et al.* 2020, and 84.6 (± 24.8) h in healthy calves; Jokela *et al.* 2024). Our results suggest that the effectiveness of meloxicam may be reduced by 48 h post-disbudding in 2–4 week -old calves, which is supported by another study showing that calves benefit from pain relief (provided via cold therapy) in the days following disbudding (Colston *et al.* 2024).

Changes in spontaneous behaviours suggest that even with meloxicam, calves may experience pain for 5 days post-disbudding (Prior *et al.* 2023), and evidence from wound sensitivity tests suggests that disbudding wounds may be painful for 9 weeks, well beyond the time-period of pain relief provided by a single injection of meloxicam (Adcock *et al.* 2018). Wound sensitivity tests found that pain relief from meloxicam may last for 3 days (Reedman *et al.* 2022), but tests for evoked pain may be differently affected by analgesics than the affective component of pain. Furthermore, Reedman *et al.* (2022) found evidence of long-term pain after disbudding when no additional meloxicam was given, suggesting that one dose of meloxicam may not be sufficient. Although how efficient and for how long meloxicam may be effective is still debated, our results suggest that calves may experience pain two days post-disbudding.

Although we expected that calves disbudded first may show a weaker aversion to the disbudding compartment than those disbudded second, we did not expect them to display a preference for this compartment neither did we expect this to be true only for Experiment 2 given that there is evidence suggesting calves find restraint and injections (which are part of the disbudding procedure) aversive (Ede *et al.* 2018; Arkangel 2023; Stillwell *et al.* 2008, and that up to 40% of calves feel pain during the disbudding procedure due to an imperfect nerve block (Thomsen *et al.* 2021). The combined action of intraoperative pain and fear of handling should have led calves to display an aversion not a preference for the disbudding compartment. This makes the lack of aversion in the calves disbudded second also surprising. However, Thomsen *et al.* (2021) did not use the needle prick test of local anaesthetic efficacy as used in this study, which could partly explain our decreased aversion for the disbudding compartment. The Bayesian analysis revealed that this absence of difference (in Experiment 1) should be interpreted carefully, so neither the null (calves did not display a preference for either compartment) nor the alternative hypothesis (calves showed a preference for a compartment) are clearly confirmed.

We do not have a full understanding of how calves remember and process events, including how levels of affective arousal and valence over time contribute to the memorisation of the experience within the 6 h of conditioning. However, it is known from rodent studies that pain relief can be perceived as rewarding and not just less ‘negative’ in rats (Navratilova *et al.* 2012). Therefore, the aversiveness of disbudding may have been counterbalanced by pain relief experienced in the hours during recovery (in contrast with what they experienced during the procedure), which may have been memorised positively, reducing the aversion for the disbudding compartment. This means that calves may have indeed found disbudding itself aversive despite the lack of aversion in the calves disbudded second in Experiment 2, and potentially explaining a stronger order effect in Experiment 2 compared to Experiment 1. In the latter, calves were only conditioned for the post-operative period, removing the contrast between the procedure and subsequent time as a potential contributor. To determine whether the procedure is aversive to calves, future trials should consider conditioning calves for the duration of the disbudding procedure only.

Another explanation may come from social buffering and emotional contagion effects since calves were conditioned in pairs. Although not well known in calves (Nogues *et al.* 2023), some recent results suggest disbudded calves are averse to seeing another calf being disbudded when they have previously been disbudded themselves (Ramirez Montes de Oca *et al.* 2024). Therefore, in Experiment 2 where calves witnessed disbudding, those who witnessed this in the second conditioning (control), having previously been disbudded, may have found this aversive which could contribute to the stronger order effect observed in Experiment 2. We chose to condition in pairs to increase statistical power, and to eliminate social isolation, which is likely aversive (Færevik *et al.* 2006). Social isolation could have reduced the contrast between conditioning treatments, limiting the effects we could detect. Little is also known about how social isolation interacts with pain perception.

### Study limitations

Our results should be interpreted with caution due to several limitations. First, the levels of pain that CPA can detect is unknown, so our results do not provide definitive evidence that calves are not in pain in the hours post-disbudding when local blocks and analgesics are used. Studies using either multimodal pain control for castration in calves (Ede *et al*. 2022), or local anaesthetic in piglets (Ede *et al*. 2024) also failed to show conditioned aversion, which may be explained by pain control strategies bringing pain to a level that is not detectable using CPA. Finally, the order effect detected in Experiment 2 was based on the time spent in the compartments during testing but was not confirmed by which compartment calves chose to enter first nor where they chose to lie down. The latter measures are inconsistently used in CPA studies (in comparison to time spent in compartments) and may be more sensitive to positive affective experiences (Lafon *et al.* 2024) or when animals are given more time to lie down (9 out of 37 did not do so in our study). Future studies could add a negative control to account for repeated conditionings (between individual design) however this design also has some limitations (e.g. increased sample size).

## Animal welfare implications and conclusion

Learning aversion/preference paradigms are an emerging method to investigate affective experiences of animals subjected to routine aversive procedures. This study builds on the growing body of literature providing a stronger understanding of animal emotions and pain. Specifically, we have applied CPA to the affective experience of calves during and in the hours following disbudding. Our results suggest that local nerve block and analgesic are sufficient in reducing pain during disbudding and in the 6 h post-disbudding to a non-detectable level using CPA. However, in Experiment 2, calves disbudded first showed a preference for the disbudding compartment, implying that these calves were in more pain 2 days post-disbudding when pain relief had worn off. Future research should confirm this result and investigate for how long calves feel in pain after disbudding.

## Supporting information

10.1017/awf.2026.10082.sm001 Ledger et al. supplementary material Ledger et al. supplementary material

## Supplementary material

The supplementary material for this article can be found at http://doi.org/10.1017/awf.2026.10082.
