# Supplementary material for: Calves disbudded with local nerve block and analgesic show conditioned place aversion two days later but not in the hours post-disbudding
Source: Anim Welf. 2026 Apr 20;35:e25. doi: 10.1017/awf.2026.10082 (PMC13101027; doi:10.1017/awf.2026.10082)
Supplement: Ledger et al. supplementary material [file S0962728626100827sup001.zip › Supplementary Figures.docx]

**Supplementary Figures**


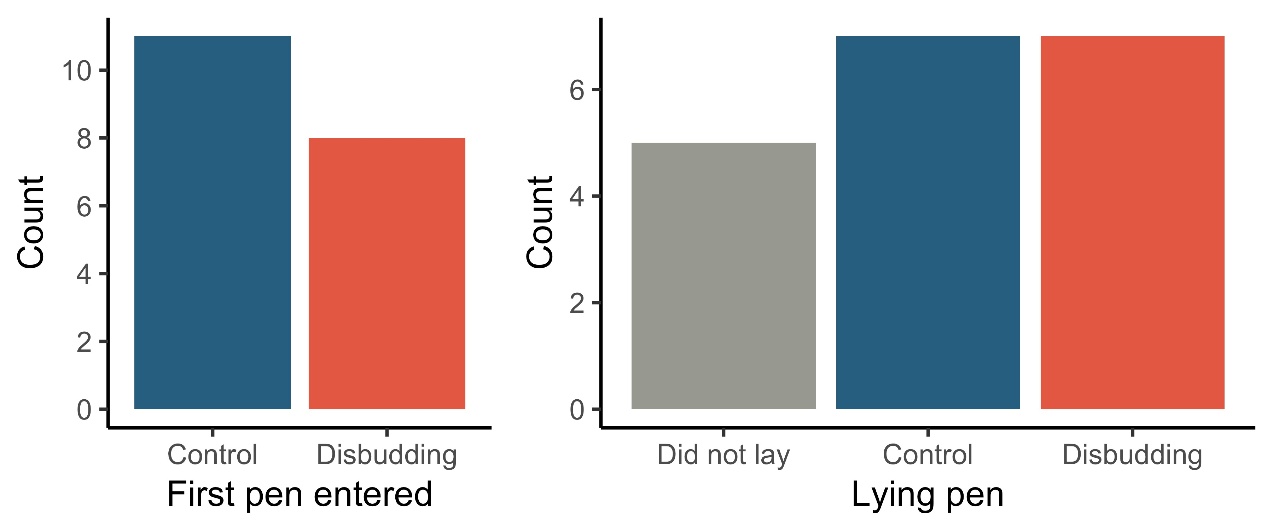


*Supplementary Figure 1. Occurrences of calves entering the disbudding or control compartment first (A), and where calves chose to lay down (B) during Experiment 1. Calves were conditioned in each compartment for a separate treatment, either for the 6 hours post-disbudding, or for a control treatment. Preference was then assessed by testing the calves 1 day after the second treatment.*


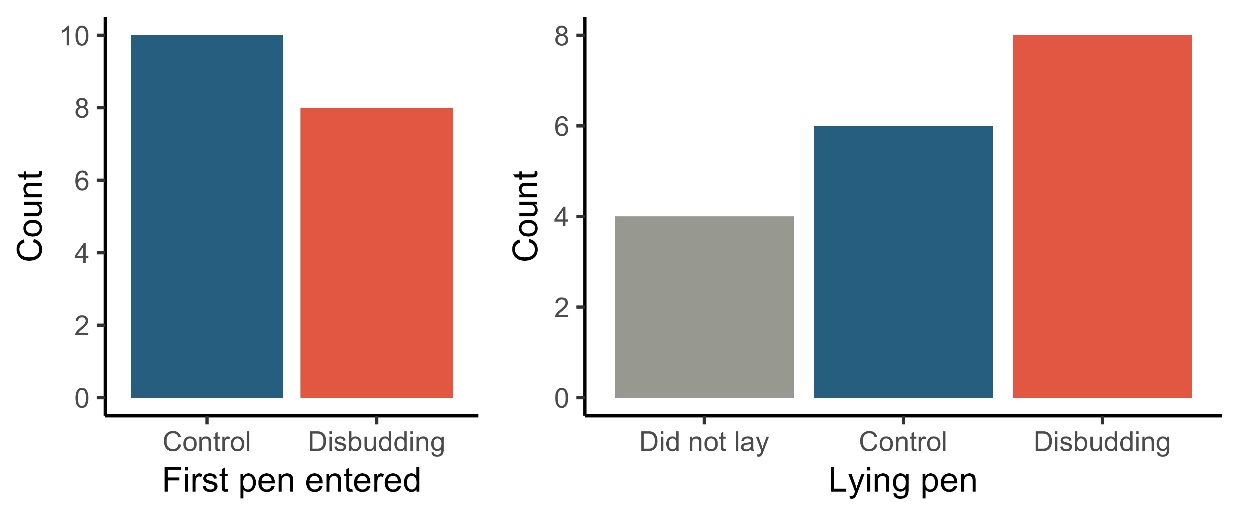


*Supplementary Figure 2. Occurrences of calves entering the disbudding or control compartment first (A), and where calves chose to lay down (B) during Experiment 2. Calves were conditioned in each compartment for a separate treatment, either for disbudding and 6 hours post-disbudding, or for a control treatment. Preference was then assessed by testing the calves 1 day after the second treatment.*
